# Long-term effects of Abay River flow regulation at Lake Tana on the geomorphic and ecological responses of the downstream river channels, Upper Blue Nile basin, Ethiopia

**DOI:** 10.1016/j.heliyon.2024.e40223

**Published:** 2024-11-07

**Authors:** Chalachew A. Mulatu, Goraw Goshu Yemer, Wubneh Belete Abebe, Yonas Amsalu

**Affiliations:** a Faculty of Civil and Water Resources Engineering, Bahir Dar Institute of Technology, Bahir Dar University, Bahir Dar, Ethiopia; b Blue Nile Water Institute, Bahir Dar University, Bahir Dar, Ethiopia; c College of Agriculture and Environmental Sciences, Bahir Dar University, Bahir Dar, Ethiopia

**Keywords:** Abay River, Blue Nile Basin, Ecohydrology, Flow regulation, Geomorphology, Lake Tana

## Abstract

Alluvial rivers adjust their geometry in response to environmental and anthropogenic disturbances. Flow regulation results in a new geomorphic condition that affects the aquatic ecosystem and its nature. This paper examines the long-term effects of flow regulation on the geomorphic and ecological responses of the downstream river channels of the Abay River, which is the only natural outflow of Lake Tana, Ethiopia. Since 1996, the river's discharge regime has been affected by constructing a head-rise weir (Chara Chara) at its natural outlet for hydropower production and dam construction on the Lake Tana tributary rivers. Hydrologic data collected at the outlet of the river, SPOT, and Google Earth images were used for the study. River banks and bed topography were extracted via ArcGIS for selected study periods. The study revealed that existing water resource development on the tributary rivers of Lake Tana modified (decreased) the outflow discharge on the Abay River, and resulted a changed morphological and moderate level ecological impact on the river system. Future ongoing and planned water resource developments will exacerbate the pressure on the lake. Without careful management, these changes are likely to have severe morphological, ecological and social consequences.

## Highlights

• The development of water resources projects around Lake Tana affects the outflow discharge of the Abay River.
• The Abay River flow regime change resulted morphological and ecological impact on the river system.
• The riverine ecosystem has moderate level of degradation.
• There are more factors than flow alteration that controls the riverine biological system.
• Maintaining the environmental flow requirement of the Abay River system is essential.

## 1 Introduction

Humans and rivers have a profound relationship with historical interactions through which civilizations grew and decayed along rivers as a result of natural and human influences [1]. It has been noticed by different researchers that anthropogenic activities and climate change are primary elements affecting rivers’ hydrological cycle [2,3]. Human activities heavily influence the nature of river flow and sediment movement, the use of land and water bodies, land cover, and river plan and bedforms; and it may result in changed hydro-morphological [4,5] and aquatic ecosystem conditions [[6], [7], [8]]. Hence, the analysis of river system changes due to flow regulation is vital to minimize the negative effects, even though it is challenging to disentangle the impact due to anthropogenic activities [9,10].

The Lake Tana basin of the upper Blue Nile (Ethiopia) has huge land and water potential for irrigation and hydropower [11]. Different medium- and large-scale projects are constructed or under construction that affect the flow to Lake Tana, the source of the Blue Nile River. The lake level was regulated by the Chara Chara Weir built in 1996 to develop two hydroelectric stations (Tis-Abay I and II) [4]. There has been an inter-basin water transfer to the Beles basin since May 2010 for the Tana Beles hydropower development [12]. An intake structure was constructed on the north-western shore of Lake Tana to take water for the Beles hydropower project to produce 460 MW of power [4].

The operation of these water resources development projects affected the Lake Tana water volume and the Abay River outflow discharge, which is the natural outflow for Lake Tana. For example, due to Tana Beles hydropower project operation, the outflow pattern of the Abay River was altered, in which over 70 % was discharged to the Beles River while the remaining was drained into the Abay River. This inter-basin water transfer for power production resulted in an increased Beles River discharge, with an average yearly release of 92 m^3^/s. This creates a hazardous situation for local communities, including the drowning of over 250 people in the river and the loss of agricultural land due to river bank erosion, which affects their food security [12]. The operation of the Beles hydropower project also affected the power production capacity of Tis-Abay I and II and, the discharge to Tis Issat Water Falls [13], both located on the course of the Abay (Blue Nile) River.

Flow regulation and water withdrawal from Lake Tana may affect the morphology of the Abay River channel. As indicated by different authors on different river systems, including Petts [14], Petts [15], Williams and Wolman [5], and Brandt [16], these interventions resulted in an altered hydro-morphology and ecology downstream of the river system. Consequently, this study analyzed the river channel adjustment and ecological consequences of the Abay River caused by water resource developments on Lake Tana and its major tributary rivers. It includes an analysis of (i) the effects of water resource developments on the discharge characteristics of the Abay River, (ii) the plan and bedform changes of the Abay River channel near the outlet, and (iii) the ecological consequences of interventions on the downstream river system. The hydro-morphological and ecological regime change in the study area may have a significant impact on the livelihoods of river-dependent communities. The alteration of natural river discharge from the previous level will affect the concentration of unwanted chemicals released to the Abay River from different industries, like the leather factory, and may affect the existence of aquatic life.

The knowledge of the river's morphology, riverine ecosystem, and its progressive change is the key to understanding the effects of ongoing climate changes and anthropogenic effects on the functioning of the river and floodplain. Acquiring such knowledge is crucial as it: (i) allows exploring relationships between the control variables (discharge and sediment load) and the response variables (channel and ecological characteristics); (ii) supports the evaluation of human intervention effects on the river hydro-morphology and ecology; and (iii) provides the basis for prediction of the future evolution of the riverine morphology and ecosystem due to planned interventions [5]. This is because assessing the impact of water resource developments required an exhaustive description of the pre-intervention and the intervention eco-hydrological and morphological characteristics of the river to assess mitigation strategies.

The study uses a combination of methods, including the analysis of historical Google Earth images and high-resolution satellite imagery (SPOT), hydro-morphological data analysis, and field observations. The successive satellite images were used to analyze the river plan and bedform changes, while the discharge analysis was used to investigate the characteristics of Abay River flow due to Lake Tana level regulation and other water resources development. This work will serve as an introduction to eco-hydrology and morphology studies along the Abay River and ecological surveys for future planned works.

## 2 Study area and data sources

### 2.1 The Lake Tana basin

The Lake Tana basin is characterized by flat to very gently sloping terrain bordering the lake on the north and east, with an area of 15,114 km^2^ [17]. The south of the basin is characterized by rolling, hilly uplands. Tropical high-land monsoons dominate the climate of the Lake Tana sub-basin. It is characterized by four seasons: (i) the main rainy season from June to August; (ii) the less rainy season from September to December; (iii) the dry season from October to February; and (iv) the minor rainy season from March to May.

The basin is also characterized by temporal and spatial climate variability. The intertropical convergence zone movement controlled the rainy season of the area, which created a single rainy season, mainly from June to September. The Lake Tana Basin receives 1,326 mm of mean annual rainfall with maximum and minimum values of 1,600 mm and 1,200 mm in the southern and northern parts of the watershed, respectively [18]. 70–90 % of the annual rainfall occurs from June to September [19], reaching its maximum in July or August (250–330 mm per month) [20]. Crop production in the area is supplemented with irrigation, as other months received little rainfall [21].

Lake Tana is located in the north-west of the Ethiopian highlands. It is the largest lake in Ethiopia and the third largest in the Nile Basin [22]. Numerous seasonal and four perennial rivers, namely, Megech, Ribb, Gumara, and Gilgel Abay, fed the lake [4,23]. The lake area is 3042 km^2^ at an elevation of 1786 m a.s.l. with mean and maximum depths of 9.53 m and 14 m, respectively [24]. Lake Tana supports various economic activities, including transportation, lakeside agriculture, and water supply for domestic and industrial use, power generation, sand mining, fishing, etc. [4,17,25].

### 2.2 Water resources developments

**Fig. 1:**
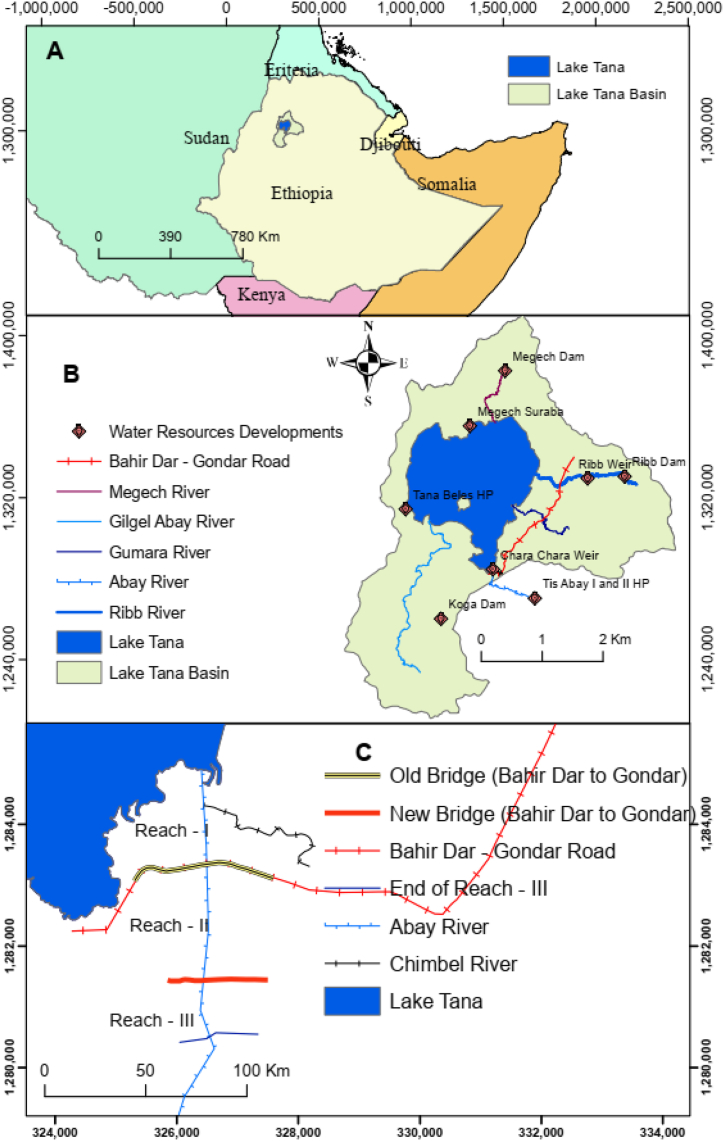
Location map of the study area. (A) Ethiopia with neighbouring countries; (B) major water resource developments in the Lake Tana basin; and (C) the study river reach of the Abay River.

The Lake Tana basin (Fig. 1B) and the Beles Basin are located in the western part of Lake Tana and the north western part of Ethiopia (Fig. 1A). The basins are considered as one of the growth corridors by the Ethiopian government [11]. Hence, several hydropower and irrigation projects have been constructed or are currently under construction.

The Tis Abay-I hydropower plant, which was commissioned in 1964 with an installed capacity of 1.4 MW on the Blue Nile River close to the Tis Issat Fall, 35 km downstream from Lake Tana, was the first in the basin [4]. The construction of the Chara Chara Weir at the Blue Nile River outlet increased the dry season river flow, maintaining the water level between 1,784 and 1,787 m above sea level, allowing Lake Tana to store approximately 9,100 million cubic meters of water [4,18]. This allowed for the construction of another hydropower plant (Tis Abay-II), built 100 m downstream of Tis Abay-I and commissioned in 1996, with an installed capacity of 72 MW [4,18]. The other is the Tana Beles hydropower plant, located on the western side of the lake, and it has been operational since May 2010. It is an artificial outlet from Lake Tana and withdraws 160 m^3^/s of water (maximum) to the Beles River to produce 460 MW of power [12,18,25]. The plant withdraws 77 m^3^/s when operated with an average plant factor of 48 % [18].

Major irrigation projects constructed in the Lake Tana basin include the Koga Dam, which stores 83 million m^3^ of water and irrigates 7,000 ha of command area [26]; the Ribb Dam, which stores 234 million m^3^ of water and irrigates 15,000 ha of command area [25]; the Megech Dam, which irrigates 7,300 ha of command area with a reservoir volume of 181 million m^3^; and the Megech Seraba pump project, which irrigates 4,000 ha of the Dembia Plain [27], located in the northern part of the lake, by extracting water from Lake Tana (Fig. 1B).

### 2.3 The study river reach (Abay River)

The Abay River is located on the south-eastern coast and contributes 8 % to the Nile River flow [28]. The river has a 40-m-high waterfall (called Tis Issat Fall) at a distance of 35 km downstream from Bahir Dar town. It is one of the most important tourist destinations (both domestic and foreign) [4]. It blocks fish migration from the lower to the upper part of the Abay River and, is considered the reason for the existence of around 20 endemic fish species in Lake Tana [29].

The study river reach for geomorphic analysis extends from the outlet of the river from Lake Tana (Chara-Chara Weir) to the downstream of a newly constructed bridge, which has a length of 4.36 km as the lower part of the reach is dominated by rock formation (Fig. 1C). It was divided into three parts (reaches) based on the existence of hydraulic structures. The first reach (Reach-1) covers a length of 1.37 km and extends from the Chara Chara Weir to the old existing bridge of the Abay River that connects Bahir Dar and Gondar towns. This reach has a tributary called the Chimble River that joins 300 m below the Weir from the north-eastern part of the river. It is a gravely dominated perennial river that originates from the upper side of a small town called Zenzelma (Fig. 1C). The second reach (Reach-II) has a length of 2.01 km and extends from the old bridge to the newly constructed bridge of the Abay River, both of which connects Bahir Dar and Gondar towns. While the third reach (Reach-III) has a length of 0.98 km and stretches from the newly constructed bridge to the end of the study river section. However, the riverine ecosystem analysis was done from the Chara Chara Weir (Abay River outlet) to the Tiss Issat waterfall.

### 2.4 Data sources

The study requires data from both primary and secondary sources (Table 1). The primary data comprises the Abay River daily discharge near the outlet of Lake Tana and the Lake Tana water level, both collected by the Ministry of Water and Energy (MoWE) of Ethiopia, and it was limited to 2015 as there is no updated data from the provider. SPOT satellite and Google Earth images were also collected to study the river morphological charges. The image acquisition date was selected based on the availability of cloud-free satellite images of the study area. Literature review was used to collect the average monthly water discharge of the tributary rivers to Lake Tana and assess different water resources projects (constructed or under construction) that may affect the Abay River outflow discharge that are, and presented in Table 2.

**Table 1:** Required data and their sources.

Data Type	Acquisition date	Resolution	Sources
SPOT Images	02 Dec. 20, 2012	2.5 m by 2.5 m	Airbus Defense and space
Google Earth Maps	06 Mar. 2005, 18 Feb. 2010, and 23 Feb. 2015		https://earthexplorer.usgs.gov/
Stream flow data	1960–2015	Daily	Ministry of Water and Energy
Lake Tana water level at Bahir Dar Station	1985–2015	Daily	Ministry of Water and Energy

**Table 2:** Periods of outflow for the Abay River at different interventions and their description based on McCartney et al. [4], and recent developments.

No	Period	Development	Description
1	1985–1996	Pre-intervention	No flow regulation of Abay River outflow from Lake Tana.
2	1997–2000	Intervention one (I-1)	Abay River outflow has been regulated by two gates of Chara Chara Weir since May 1996 to produce power at Tis Issat [4].
3	2001–2010	Intervention two (I-2)	Abay River outflow from the lake was regulated by constructing an additional five new gates at the Chara Chara Weir since January 2001 to produce power at Tis Issat I and II [4].
4	2010–2015	Intervention three (I-3)	In this period, different water resource projects are developed: 1. The Tana Beles hydropower plant has been operational since May 2010. It transfers 160 m^3^/s of water from the lake to the Beles River to produce 460 MW of power [18]. 2. Koga irrigation project, commissioned in 2008, in the Koga River, to irrigate 7000 ha [26].
5	After 2015		1. Megech Seraba pump irrigation project withdraws 30.7 million m^3^ of water from Lake Tana to irrigate around 4,000 ha of command area [27]. 2. Ribb Dam on the Ribb River that stores 234 million m^3^ of water and irrigates 15,000 ha [25]. Even though the dam part is inaugurated on October 28, 2018, the project is not operational since now as the construction of irrigation system is not finalized. 3. Megech dam on the Megech River. It is under construction to store and irrigate 181 million m^3^ of water and 7,300 ha of land, respectively [27]. It is under construction.

## 3 Materials and methods

### 3.1 Abay River flow regime characterization

More than 93 % of the Lake Tana inflow originates from four major rivers, namely Ribb, Gumara, Gilgel Abay, and Megech [20]. The Abay River flows towards the eastern corner of Bahir Dar City at the southern end of the lake. The river flows down for 35 km in a south-east direction until it forms the attractive Tis Issat waterfall, dropping into a 40-m gorge, a famous tourist destination [29].

The discharge analysis of the Abay River was carried out considering the major water resource developments in the Lake Tana Basin that significantly affect the river's outflow (discussed in Section 1.2). Hence, the analysis period was divided into two major categories: the pre-intervention and intervention periods. The pre-intervention represents the period before April 1996, in which there were no major water resource developments around Lake Tana that significantly affected the Abay River discharge (Table 2). The period represents the natural inflow and outflow conditions of Lake Tana and can be considered a baseline to study the hydro-morphological and ecological changes of the river. In this period, the river discharge is dependent on the rainfall characteristics and run-off of the watersheds. The discharge data required for the study was collected from the Ministry of Water and Energy (MoWE).

In the intervention period (after 1996), different water resources projects were developed for hydropower and irrigation (Section 1.2), all of which reduced or altered the outflow discharge of the Abay River. The period considers the operation of the Chara Chara Weir, the Tana Beles hydropower development, and the construction and operation of dams on the tributary of Lake Tana and divided into four based on interventions that affected Abay River discharge (Table 2).

Long-term discharge characterization is used to identify and evaluate the overall flow regime change and its effect due to different levels of discharge regulation. This was done based on the discharge values of the nearest gauging station, downstream from the Chara Chara Weir, for the pre-intervention and intervention periods. The discharge regime change due to water resource developments was examined based on the time series measured discharge hydrographs of the mean monthly covering the period 1973–2015 even though irrigation projects on the tributary rivers that may affect the outflow discharge were constructed (under construction) as shown in Table 2. The computed flow parameters also include the annual peak and lowest flows. Knowledge of minimum flow values and their duration is essential to characterizing the in-stream and riparian ecosystems.

The bankfull discharge is an important reference condition to study the river flow and morphology [30]. It can be computed using stage-discharge rating curves and flow frequency analysis of the annual maximum series [31]. However, due to a lack of river geometry and water levels at the gauging station, flow frequency analysis based on Gumbel extreme distribution (Eq. (1)) was applied by filtering the annual maximum of each year from the daily discharge time series [32]. The method assumes that the annual peak and the instantaneous peak discharge have equal values for each year and the discharge that occurred with a return period of 1.5 and 2.0 years are representative for the bankfull conditions [33]. It was applied by Petit and Pauquet [34] to study the bankfull discharge of different rivers with a catchment area varying from 4 km^2^ to nearly 2700 km^2^.

(1) $$Q_{T}=Q_{avg}+K_{T}\times \sigma$$

in which Q_T_ is the discharge magnitude (m^3^/s) having a return period of T years, Q_avg_ is the average value of the annual peak flows (m^3^/s), and σ is the standard deviation of the flows (m^3^/s). The dimensionless frequency factor (K_T_) is given by Eq. (2), written as:

(2) $$K_{T}=\frac{\sqrt{6}}{\pi }\left\{\lambda +\ln\left[\ln\left(\frac{T}{T-1}\right)\right]\right\}$$

where λ is the Euler constant (=0.5772) and T is the return period in years.

The Gumbel extreme method may result in some uncertainty when computing bankfull discharge values due to the limited number of samples [32,35]. Hence, we calculated the confidence interval that indicates the range between which the true value may exist with a specific probability based solely on sampling errors. The upper (+) and the lower (−) confidence interval for the mean value of the bankfull discharge was computed using Eq. (3) below.

(3) $$C_{I}=Q_{aveg}\pm E$$

Where C_I_ is the confidence interval values of the bankfull discharge (m^3^/s), E is the margin of error $(=t_{a/2}\frac{\sigma }{\sqrt{n}}$), α is the significance level (=1- C_I_), t_α/2_ is the critical value (a function of the degree of freedom, which is sample size minus one), and n is the number of samples.

### 3.2 Plan and bedform analysis of Abay River

River channels are formed by the discharge they carry, the sediment supply of the watersheds, their transport capacity, and the geological formation of their beds and banks [36,37]. ArcGIS and manual onscreen was used to digitize the high-resolution SPOT satellite, available in our archive, and cloud-free Google Earth images of 2005, 2010, 2015 and 2020 (Table 1), respectively. In this way, the time-series morphological features of the river bed and banks was extracted [38]. The planform analysis of the river was then related to flow regulation, and categorized into different intervention periods (Table 2).

### 3.3 Ecological analysis

The effect of water resource developments on the release of environmental flow and aquatic life was analysing by the change in discharge (ecologically relevant minimum flow) from the pre-intervention period using IHA software [39]. The ecological status of the Abay riverine ecosystem between the Chara Chara Weir and Tis Issat fall was studied using the modified NovaWET technique (https://novascotia.ca/nse/wetland/docs/NovaWET.3.0.pdf, retrieved on 05 May 2023). Required data for the study, including the river discharge, was collected using primary and secondary sources. In addition, the relationship between the flow alteration of the Abay River and riverine vegetation was analyzed using the normalized difference vegetation index (NDVI) for a distance of 500 m buffer from the centreline of the Abay River stretching from the lake to the Tis Isat Falls [40].

## 4 Results and discussion

### 4.1 Hydrological regime change due to interventions

**Fig. 2:**
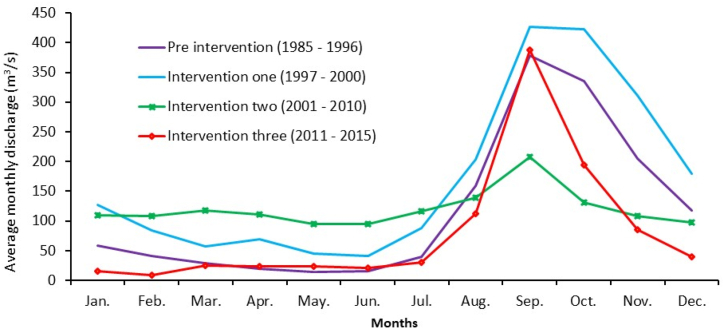
Average monthly Abay River discharge at its outlet. The graph shows the effect of flow regulation since 1997.

The pre-intervention hydrological analysis was considered a baseline to study the impacts of other water resource developments on the Abay River and on the other tributary rivers that drain to Lake Tana. In these periods (1985–1996), the maximum and minimum average monthly discharge values were 378 m^3^/s and 15 m^3^/s in September and May, respectively, while the average was 118 m^3^/s (Fig. 2). The large discharge gaps between the maximum and minimum values indicate natural seasonal river discharge variability. As noted by McCartney et al. [4], the Abay River discharge variability before the Chara Chara Weir operation was closely linked to the rainfall characteristics of the watershed. However, the construction of storage dams affects the downstream discharge hydrograph, sediment volume and river morphology as noted by Chen et al. [37] and Chen et al. [41] in the Yellow River basin.

From May 1996 to December 2000 (period of intervention one, I-1), the two gates of the Chara Chara Weir were operational for power production. The observed river discharge was significantly greater than the pre-intervention period for the wet and dry seasons, with maximum and minimum average monthly values of 427 m^3^/s and 42 m^3^/s in September and June, respectively, with a yearly average of 171 m^3^/s (Fig. 2). During these periods, the Lake Tana water level regulation by the Chara Chara Weir increased dry season discharge since the water was released for Tis Issat I and II hydropower generation [25]. The higher wet season discharge values may be related to heavy rainfall in the upper watershed areas [4].

The Chara Chara Weir was regulated for full-gate condition (Table 2) from January 2001 to December 2010 (intervention two, I-2). The maximum and minimum average monthly discharge values of the river were found to be 208 m^3^/s and 94 m^3^/s in September and June, respectively, while the average annual flow was 120 m^3^/s (Fig. 2). The Chara Chara Weir full-gate flow regulation resulted in higher and lower dry and wet season outflows, respectively. This resulted in much less seasonal flow variability in the lake, as described by McCartney et al. [4]. They found that 43 % of the discharge from the lake occurred in the five months (February to June) after 2001.

The dry season outflow discharge values of the river have shown further reduction since January 2011 (Intervention three, I-3) due to the operation of the Tana Beles hydropower plant and the irrigation system downstream beside the Chara Chara Weir. This hydropower plant transfers the water from Lake Tana to the Beles River following the creation of an artificial outlet followed by the diversion of water from Lake Tana to the adjacent basin, which has reduced the natural outlet's outflow, i.e., the Abay River (Fig. 2). The project transfers a maximum discharge value of 160 m^3^/s through a tunnel to produce 460 MW [18]. However, the wet season discharge shows an increment.

**Fig. 3:**
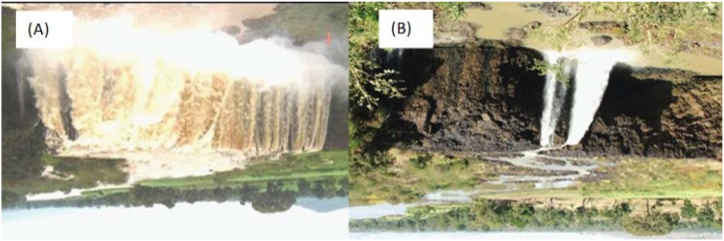
Effect of flow regulation on the Tis Issat waterfall of the Abay River, 35 km downstream from its outlet. (A) Before regulation (i.e. before 1996) and (B) after regulation [4].

Annys et al. [12] analyzed the Beles River discharge at the hydropower outlet and found an additional yearly average discharge of 92 m^3^/s, which is above the planned discharge of 77 m^3^/s [42]. This amount of discharge should be released to the Abay River if there is no regulation for Tana Beles hydropower. Due to these, a fixed release of 17 m^3^/s to the Blue Nile River outlet has been proposed, with an absolute minimum of 10 m^3^/s as environmental flow [43]. The effect of discharge regulation is clearly seen in Fig. 3A and B, where it shows the comparison of the Tis Issat waterfall in the pre-intervention and intervention periods. Another intervention in these periods was the construction of major dams in the tributary rivers of Lake Tana for irrigation, as described in Section 1.2. They all resulted in the reduction of wet and dry season discharges to Lake Tana and then to the Abay River, as all are constructed for irrigation. The effect of climate change, which affects long-term regional water resources (surface water and groundwater potentials), as noted by Khoi et al. [44] in Ho Chi Minh City, Vietnam, may also affect outflows of the river. Here, it should be noted that the result of this study is a combination of climate change and anthropogenic activities, despite the fact that Xu et al. [2] indicate that anthropogenic activities play a dominant role in influencing streamflow indicator variation and hence river morphology.

Moreover, the bankfull discharge and its change due to intervention was analyzed using the Gumbel equation as described in Section 2.1, and the results are shown in Table 3. Operation of Chara Chara Weir for all gate operations (Intervention two) resulted in a reduced bankfull discharge as it lowers the wet season peak discharge values. The percentage of time that the computed bankfull discharge equaled or exceeded the 1.5- and 2-year return periods is over 67 % and 40 %, respectively. The upper and lower limits of the 90 % and 95 % confidence intervals for the bankfull discharge were calculated using Eq. (3), and the results indicate that the values fall within these confidence intervals.

**Table 3:** Estimated bankfull discharge for different intervention periods.

Intervention	Bankfull Discharge (m^3^/s) for a return period of:	
	1.5 years	2 years
Pre intervention	258.73	315.32
Intervention one	364.75	425.20
Intervention Two	203.84	284.29
Intervention Three	437.80	462.96

### 4.2 Analysis of Lake Tana water level

**Fig. 4:**
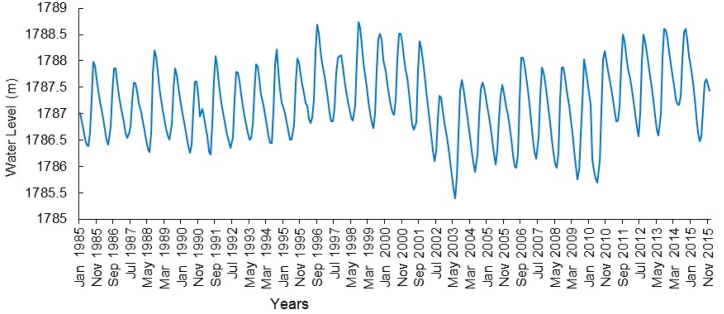
Mean annual water of Lake Tana level since 1985 (data source: Ministry of Water and Energy, Ethiopia).

Lake Tana water level regulation for power production, and the construction of major dams in the tributary rivers for irrigation affect the Abay River outflow volume and characteristics. The lake level rises slowly to reach its maximum in September (the end of the main rainy season) and recedes slowly to its minimum water level in June (Fig. 4). As reported by McCartney et al. [4], flooding and water level drops occur frequently in the area around the lake. Mulatu et al. [33] also analyzed an excessive Lake Tana level fluctuation since 1997 due to modifications of the natural lake water levels and the river flow regime. They indicated that the Lake Tana water level regulation for hydropower production resulted in a rise, a drop, and then a rise of 45, 53, and 62 cm (average) for the periods of 1995–2001, 2001–2010, and 2010–2015, respectively.

In the pre-intervention period, the average yearly Lake Tana water level variation was 1.5 m, which is lower than in the first intervention period, related to flow regulation by Chara Chara Weir. Since the operation of the Weir, the dry-time lake level has dropped dramatically, reaching the historical minimum water level of 1784.46 m a.s.l. in 2003. From 2001 to 2010, the Lake Tana water level showed a reduction due to second phase of the Chara Chara Weir operation (Intervention two). Despite this, there has been an increase in the level since 2011, due to the major shift from Tis Issat I and II to Tana Beles Hydropower Plants. During this time, the Chara Chara gates are closed to keep water in Lake Tana for use by Beles power generation. These variations have impacted the livelihoods of people living in the vicinity of both the lake and the Abay River [4].

### 4.3 Plan and bedform change of Abay River

**Fig. 5:**
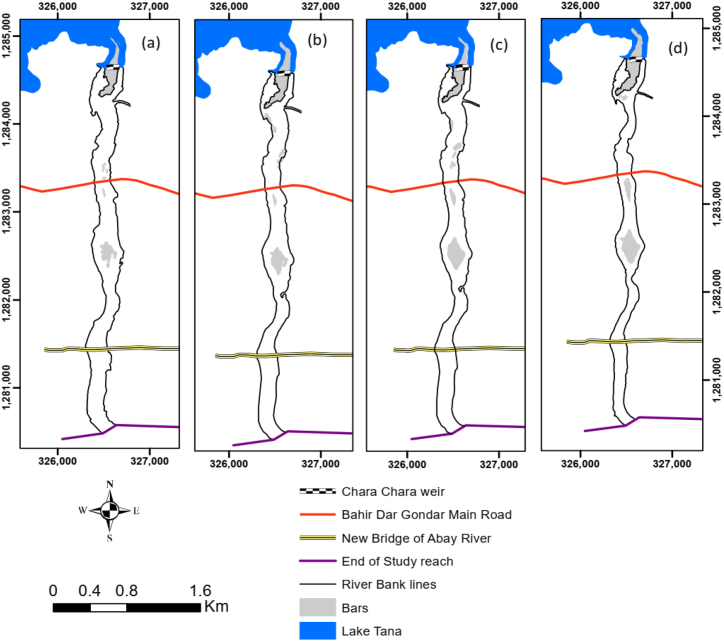
River planform change and bar developments through time. (a) 2005, (b) 2010, (c) 2012, and (d) 2015.

Analysis of river bedform using Google Earth and SPOT satellite shows that the active river channel had an area of 849 ha in 2005 and, it was reduced to 792 ha, 730 ha, and 623 ha in 2010, 2012, and 2015, respectively. This indicates that 27 % of the active river channel was lost within 10 years (from 2015 to 2005). On the other hand, the area of bars (Fig. 5a-d) has increased over time. The total bar area in 2005 was 87 ha, whereas it was 128ha in 2015, showing a 49 % increase.

The reduction of active river channels and the increment of river bars are mainly related to the decreasing outflow discharge from the Abay River due to the development of different water resource projects on the Lake Tana and its tributary rivers, as discussed in Section 3.1. In addition, there may be deposition of sediments along the river banks and in the channel as the reduced discharge may not have the power to move the incoming sediments from the watershed and tributary rivers [16].

**Fig. 6:**
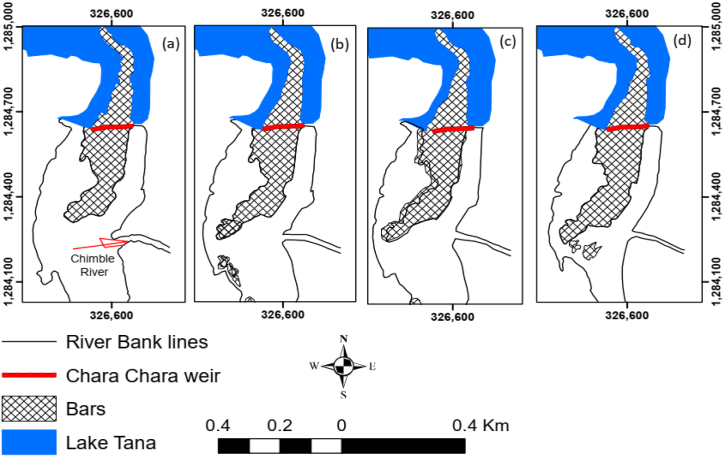
Planform change (bar development) downstream of Chara Chara Weir and near the confluence of Chimble River. (a) 2005, (b) 2010, (c) 2012, and (d) 2015.

A reduction in flooding intensity may provide favourable conditions for vegetation growth on riverbanks and bar tops. This is also true in the case of the study area, where vegetation growth on the bars and the river banks is visible nowadays. According to Tal and Paola [45], riparian vegetation varies with river discharge and sediment accumulated during high river flows will be stabilized, and more sediment will be trapped. Similar results were also found by Caruso et al. [46] on the braided Ahuriri River in New Zealand, in which vegetation growth is positively correlated with the frequency of the occurrence of high flows. Fig. 6(a–d) shows no increasing or decreasing trend in the area of bars upstream of the Chara Chara Weir, despite the existence of increased vegetation encroachment over time. Increasing phosphorus and nitrogen levels, along with degradation of other water quality parameters, have spawned an expansion of water hyacinths in Lake Tana, along the mouths of major rivers [47]. They also positively associate the expansion of water hyacinths with an increase in Lake Tana water level and flooding as the result of the Chara Chara Weir.

It is likely that the Abay River outflow discharge will not contain much sediment due to the large surface area of Lake Tana (Section 1.1). However, Fig. 6(a–d) shows an increased sandbar near the Chimble River confluence due to the inability of the Abay River outflow discharges to transport sediment supply from the perineal tributary Chimble River. The rainy season backwater along the Chimble River due to increased Abay River flow also creates a favourable condition for sediment accumulation near the confluence. Williams and Wolman [5] also indicated the occurrence of sedimentation downstream of the river channel confluence, as the regulated flow was insufficient to move the sediment. Moreover, long-term river discharge reduction resulted in bar formation, channel braiding, and conveyance capacity reduction [5].

### 4.4 Effect of flow regulation on the Riverine ecosystems

The Abbay Riverine wetland, extending from Chara-Chara Weir to Tis Issat Falls, has undergone significant anthropogenic modification due to urbanization and intensified agriculture. This alteration has affected the wetland's floodwater detention capacity, ecological integrity, and connectivity with the surrounding landscapes affecting its overall ecological resilience. Despite a growing interest in wetland conservation by federal, provincial, and local authorities, the Bahir Dar City Administration has not initiated concrete restoration or preservation efforts beyond the designation of the area as a park (Fig. 8a).

**Fig. 7:**
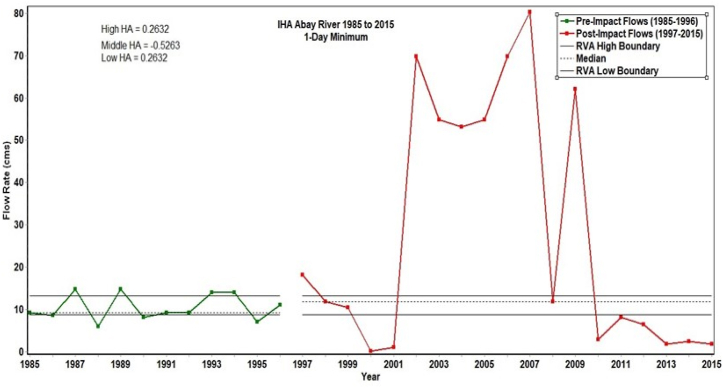
Abay River minimum flow between 1985 and 2015. Where RVA refers to Range of variability approach (Looking at before/after flow percentiles).

This study revealed that the Abay riverine ecosystem has undergone moderate degradation, primarily due to a shift in the hydrological regime and a decline in riparian vegetation. The ecologically significant low flow, which was 98.87 m³/s before 1996 (pre-intervention), dropped to 54.81 m³/s from 2009 onward (Fig. 7). The hydrological alteration (HA) values for both high and low minimum flows before and after the intervention showed positive trends, indicating increases in both. Specifically, the increase in low HA suggests a decline in minimum flow, particularly after 2009, as 5 data points fall below the median out of 7 (Fig. 7). This study indicated that the Abay riverine ecosystem has been degraded to a moderate extent, and that the major cause is the hydrological regime shift accompanied by the decline of riparian vegetation.

**Fig. 8:**
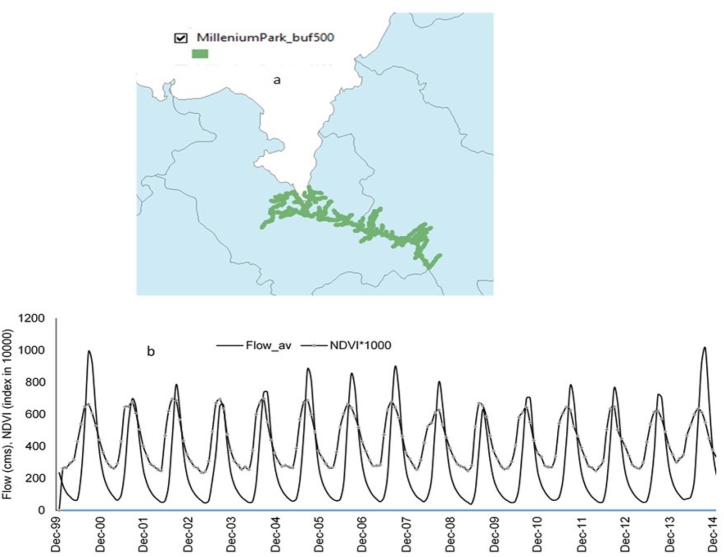
a) Study river stretch along Abay River from the lake to Tis Isat Fall. b) Monthly NDVI and flow trends from 2000 to 2015 in the Abay River for the designated stretch for Millennium Park; Note: the green-coloured area is a 500 m buffer from the river thalweg that is considered as the riparian zone for the vegetation analysis.

The study river section is also under tremendous pressure from point and diffuse pollutants, expansion of human settlements, and destruction of riparian wetland from unsustainable agriculture [17].

The riparian vegetation index increased between 2000 and 2007, decreased between 2008 and 2013, and has shown a rising trend since then (Fig. 8b). This pattern aligns with the flow conditions of the Abay River, with a Pearson correlation coefficient of 0.74, suggesting a strong relationship between riparian vegetation health and river flow. James et al. [48] and Abebe et al. [49] have reported similar findings. Although the flow conditions exhibit decadal fluctuations and a low coefficient of determination, the overall trend indicates an increase in flow. This relationship underscores the importance of vegetation in regulating hydrological processes.

Although this study did not include a sensitivity analysis of the relationship between vegetation cover and river discharge, Sun et al. [50] demonstrated that adjusting NDVI to simulate vegetation changes from reforestation or land degradation revealed a high sensitivity of river discharge to vegetation cover, particularly in headwater regions. Similarly, studies on the Nile and Congo Rivers have shown that shifts in vegetation due to agricultural activities or climate change can significantly impact river flows [51].

The discharge of untreated waste from hotels, industries, and other sources has significantly degraded the river's water quality. Notable point sources of pollution include the Bahir Dar Textile and Bahir Dar Leather industries, which release untreated or partially treated waste into the river [52,53]. Pollutants like fertilizers, sewage, industrial waste, and agricultural runoff can also adversely affect the quality of freshwater and make it unsafe for the public as well as for environmental consumption. Additionally, sediment accumulation and excessive vegetation growth have further negative impacts on the river's ecosystem.

The alteration of natural flow and water level patterns can directly stress aquatic organisms and significantly change the chemical and physical properties of aquatic and riparian ecosystems, leading to declines in native species richness, abundance, and distribution. The biodiversity in the Abay River between Chara Chara Weir and Tis Issat Falls includes two fragmented hippopotamus colonies, fish, benthic organisms, and wetland vegetation such as *Cyperus papyrus*. The hippopotamus population in the Blue Nile (Abay) River near Bahir Dar has drastically declined, with numbers dropping from 20 in 1998 to just six in 2003. The few remaining hippos struggle to survive in a disturbed habitat close to human activity, with limited food resources [54].

Changes in the river's discharge regime, which alter the natural cycles of flooding and sedimentation, affect habitats and migration patterns for species like freshwater fish and hippos. Modifications to river channels can lead to faster or slower flows, causing erosion or deposition, and complicating migration for some species. While Lake Tana is home to 26 known fish species, the fish fauna below Tis Issat is not well-studied [55]. However, Aynalem et al. [56] reported that *Labeobarbus* species were the most dominant fish (40 %) in the Abay River reach between Chara Chara and Tis Issat Falls.

The low flow conditions that go to the extent of dis-connectivity and high silt accumulation have constrained the up- and downward movements of the hippos, affect the prey-predator relationship, and affect the performance of the riverine fishes; they close the interstitial spaces and affect the soil structure, which affects the breeding and feeding grounds of fish.

Besides the above-mentioned direct impacts, river flow regulation can also result in various indirect effects, such as ecological shifts (including changes in riparian vegetation and loss of biodiversity), and reductions in downstream activities like flood-recession farming and fishing. However, communities may also benefit from the hydropower generation and the stable water supply for irrigation, contributing to economic growth [57].

## 5 Conclusion

This study examines the impact of flow regulation on the Abay River channel near its outlet at Lake Tana, Ethiopia. Time-series analysis of satellite images, discharge and lake level data was used to analyze the morphological and ecological impacts. Dry-time Google Earth images and SPOT satellite images were used to investigate channel adjustments over the last 10 years (2005–2015). The analysis was limited until 2015, as the provider (MoWE) did not update the river daily discharge and the lake water level data. This hinders our analysis to examine the effect of recent interventions and draw the relationship between anthropogenic factors, outflow discharge, and the river's morphological and ecological changes.

The study revealed that the construction of the Chara Chara Weir did not affect the Abay River's annual discharge volume, as its role is limited to regulating outflow. However, it has significantly altered the river's high and low flow rates, which are critical for maintaining the balance of the river's morphology and ecology. In contrast, the implementation of the Tana Beles hydropower project has considerably reduced the river's annual discharge and volume, leading to lower flow levels during both wet and dry seasons. Additionally, other water resource developments on the tributaries of Lake Tana have impacted the river's flow regime, forcing the Abay River to adjust its channel dimensions, which has led to new hydro-morphological and ecological changes.

The ecological conditions of stream corridors depend on river hydrological conditions, morphological processes, catchment geology, topography, climatology, vegetation cover, land use practices, and human interventions. Understanding the morphological and hydrological characteristics of a catchment is essential for assessing its environmental health and predicting the effects of management and development activities on the river and surrounding ecosystems.

In the future, environmental flow estimates and comprehensive studies should be conducted to assess the river's ecological sensitivity to flow modifications, from the Lake Tana outlet to Tis Issat Falls and further downstream. Identifying priority areas within the sub-basin and implementing proper water allocation and regulation mechanisms according to these priorities is critical. Current water management practices in the sub-basin are likely insufficient to mitigate the negative impacts of climate change on sedimentation, flood risks, energy production, and aquatic ecosystems. This study did not also investigate the specific morphological and ecological changes resulting from anthropogenic impacts and climate change, both of which are major factors influencing the watershed's hydrology.

Upland watershed conservation is key to preserving the river's natural base flow, and efforts should be made to restore the Abay River's natural watercourse. To address the impacts of river regulation, restoration initiatives should focus on re-establishing dynamic connectivity between the Abay River channel and floodplain water bodies.

## CRediT authorship contribution statement

**Chalachew A. Mulatu:** Writing – review & editing, Writing – original draft, Methodology, Investigation, Formal analysis, Conceptualization. **Goraw Goshu Yemer:** Writing – review & editing, Writing – original draft, Methodology, Conceptualization. **Wubneh Belete Abebe:** Writing – review & editing, Methodology, Investigation, Conceptualization. **Yonas Amsalu:** Writing – review & editing, Writing – original draft, Methodology.

## Ethical statement

The authors state that the research was conducted according to ethical standards.

## Data availability statement

All relevant data are included in the paper or its Supplementary Information.

## Declaration of competing interest

The authors declare that they have no known competing financial interests or personal relationships that could have appeared to influence the work reported in this paper.
